# Assessing cytotoxicity and endoplasmic reticulum stress in human blood–brain barrier cells due to silver and copper oxide nanoparticles

**DOI:** 10.1007/s13353-024-00833-8

**Published:** 2024-02-09

**Authors:** Luiza Chojnacka-Puchta, Dorota Sawicka, Lidia Zapor, Katarzyna Miranowicz-Dzierzawska

**Affiliations:** Central Institute for Labour Protection - National Research Institute, Czerniakowska 16, 00-701 Warsaw, Poland

**Keywords:** Copper oxide nanoparticles, Cytotoxicity, Endoplasmic reticulum stress-induction, Human cerebral microvessel endothelial cells, Silver nanoparticles

## Abstract

In recent years, it has been generally accepted that metal-based nanoparticles (NPs) may induce stress in the endoplasmic reticulum (ER), a key organelle where protein folding occurs. We examined ER stress in immortalized human cerebral microvascular cells (hCMEC/D3) after exposure to silver-NPs (Ag-NPs)- and copper oxide-NPs (CuO-NPs) induced toxicity at < 10 nm and < 40 nm or < 50 nm diameters, respectively. In cytotoxicity assessments, cells were exposed to different CuO-NPs (5–400 µg/mL) or Ag-NPs (1–10 µg/mL) concentration ranges for 24 h and 72 h, and tetrazole salt reduction assays (EZ4U) were performed. Also, Ag-NP or CuO-NP effects on cell proliferation, apoptosis (caspase 3/7 assays), and ER stress and cell morphology were evaluated. In ER stress assessments, RNA-like endoplasmic reticulum kinase (PERK), activating transcription factor 6 (ATF6), inositol-requiring enzyme 1 (IRE1a), and others stress factor mRNA levels were determined after 24 h treatment using Real-Time PCR. Increased stress sensors (IRE1a, PERK, and ATF6) mRNA levels were observed after exposure to Ag-NPs (< 10 and < 40 nm) or CuO-NPs (< 50 nm). We investigated the expression of tight junction (TJ) proteins (barrier junctions) and showed that both types of NP reduced of OCLN gene expression. Morphological changes were observed after Ag-NP or CuO-NP exposure using holotomographic microscopy. Our data suggest that Ag- and CuO-NPs should undergo future in vitro and in vivo toxicology studies, especially for downstream biomedical application and occupational risk assessments.

**Supplementary Information:**

The online version contains supplementary material available at 10.1007/s13353-024-00833-8.

## Introduction

Nanomaterials are unique molecules widely used in many biomedical applications (Gim et al. 2019; Mabrouk et al. 2021; Díaz-Puertas et al. 2023). Due to the biological advantages and distinct functions of copper (Cu) and silver (Ag) nanoparticles (NPs), new biomaterials containing Ag-NPs and Cu-oxide-NPs (CuO-NPs) have been developed (Mousa et al. 2020; Calabrese et al. 2021; Diez-Pascual and Rahdar 2022). Both elements are widely used to promote wound healing, function as innovative antimicrobial agents, and support tissue and bone regeneration (Chowdhury et al. 2016; Burdușel et al. 2018; Kraeling et al. 2018; Wang et al. 2020).

Due to its unique properties, Ag is extensively used in the cosmetics (creams and ointments) (Ong and Nyam 2022) and medical device (catheters and dressings) industries (Burdușel et al. 2018). However, in previous work, Zapor (2016) examined the toxic effects of differently sized Ag particles (~ 10, 40, and 100 nm) in reproductive and respiratory system cells and reported that Ag-NPs, especially those < 10 nm, posed serious threats to cells. Tang et al. (2009) and Sharma et al. (2009) also reported that intravenous or subcutaneous Ag-NP injections disturbed the blood–brain barrier (BBB), caused astrocyte swelling, and degenerated rat neurons.

Nano-CuO is an antiviral material used to prevent infectious diseases and can be incorporated into personal protective gear and other healthcare products (Ingle et al. 2014). CuO-NP is also used in pesticides and as a nutritional supplement in cattle and poultry feeds (Parada et al. 2019; Adisa et al. 2019). Similarly, nano-catalysts, based on Cu and Ag, have gained considerable traction in catalytic applications, organic transformations, and electrocatalysis and photocatalysis (Gawande et al.; 2016; Liao et al. 2019). CuO-NPs have been incorporated into matrix materials during nanocomposite fabrication due to their large surface-to-volume ratios, antibacterial surfaces, and electrical applications (Din and Rehan 2017).

The literature has reported that Ag- and CuO-NP interactions with cell membranes or individual organelle are extremely important and require toxicity assessments, especially considering long-term exposure consequences. Excess cellular Cu causes organelle damage and elicits toxic effects (Feng et al. 2022). Thus, in biomedicine, determining ER stress levels mediated by NPs in endothelial cells is critical (Kusaczuk et al. 2018; Khan et al. 2020; Zheng et al. 2023).

Cell exposure to NPs can impair correct ER functioning and specifically accumulate misfolded proteins in the ER (Zhang et al. 2016; Onoda et al. 2020) consequently, cells activate ER stress responses where ER chaperones are released and mRNA translation blocked to reduce protein synthesis and ER load (Yoshida et al. 2000). The Unfolded Protein Response (UPR) mediates cell responses to ER-associated stress and helps restore homeostasis by increasing chaperone protein expression (including BiP and GRP94) (Schröder and Kaufman 2005), avoiding cytotoxic effects caused by the impaired recognition of unfolded proteins, and releasing factors to inhibit translation (Adams et al. 2019). However, UPR activation and prolonged ER stress can initiate apoptosis in cells (Tabas and Ron 2011; Sano and Reed 2013; Liu and Tang 2020).

During altered protein processing and excessive unfolded protein accumulation, ER stress dissociates BiP from effector proteins in the ER membrane, including protein kinase RNA-like ER kinase (PERK), inositol-requiring protein 1 (IRE1), and transcription factor 6 (ATF6) (Hou et al. 2013; Huo et al. 2015). ER stress activates three major protein-related pathways, where proteins become active and dimerize to induce different genes to either restore normal ER function or induce apoptosis. IRE1 induces alternative splicing of the X-box binding protein 1 (XBP-1) transcription factor. ATF6 is then phosphorylated, and with XBP-1, induces the transcription of UPR genes encoding several chaperones. PERK blocks protein translation by phosphorylating the translation eukaryotic initiation factor-2α (eIF2α) (Brown et al. 2014; Christen and Fent 2012). ER stress is responsible for the development of many diseases in the human body, which is important in toxicological assessment, and in particular, ER stress should be considered as an early biomarker in nanotoxicological studies (Huo et al. 2015; Simard et al. 2016; Yang et al. 2017; Liu and Tang 2020).

Assessing NP interactions with endothelial barrier cells is important as these cells are implicated in tissue and organ homeostasis, barrier maintenance, and healing processes (Chistiakov et al. 2015; Aman et al. 2016; Claesson-Welsh et al. 2021). A unique feature of brain vascular endothelial cells is their elevated expression of tight junction (TJ) proteins (barrier junctions) or simply TJs. Li et al. (2015) reported that Au-NPs increased both in vitro pericellular endothelial permeability and in vivo BBB permeability. They observed that Au-NPs induced endothelial TJ instability and caused proteasome-mediated TJ degradation. Similarly, NP interactions affected BBB structures; endothelial cells lost their protective effects against toxic substances. However, NP effects on the BBB and their mechanisms of action remain largely unclear. By studying NP-mediated ER stress in BBB cells, important insights on NP-related toxicity can be generated and provide guidance on NPs as anti-disease therapeutics. Different BBB cell models have been used in in vitro studies; most are mouse and rat based, but some are double or triple brain vascular endothelial cell, pericyte, and astrocyte co-cultures (Williams-Medina et al. 2021; Shah and Dong 2022; Pérez-López et al. 2023). Critically, Xu et al. (2015) observed TJ disruption, decreased TJ expression, and increased BBB permeability after 24 h exposure to Ag-NPs in triple co-culture rat BBB cells.

In this study, we examined in vitro ER stress and cytotoxicity due to Ag-NPs and CuO-NPs exposure in an immortalized human cerebral microvascular cell line model (hCMEC/D3). We investigated real time in vitro functional and morphological changes in cells and early changes in cell responses after NP exposure.

## Materials and methods

### NP characterization using Dynamic Light Scattering (DLS)

Commercially available NPs were purchased from Sigma-Aldrich Chemical Co. (St. Louis, MO, USA; CAS Ag-NPs < 10 nm No. 730785, Ag-NPs < 40 nm No. 730807, and CuO-NPs < 50 nm No. 544868-5G). All particles were subjected to TEM analysis and spectral property evaluation, and characterized in terms of diameter and size distribution by the manufacturer Sigma-Aldrich. Both Ag-NPs < 10 nm and < 40 nm were defined as colloidal NPs of diameter = 10 ± 4 nm/40 ± 4 nm at 0.02 mg. CuO-NP (< 50 nm) stock solutions were prepared in phosphate-buffered saline (PBS, Gibco, Invitrogen, Carlsbad, CA, USA) at 1 mg/mL. Suspensions were sonicated at room temperature at 1.4 kJ/cm^3^ for 30 s and 90% amplitude (Sonica Q 700, Qsonica LLC, USA).

NP hydrodynamic diameters were measured in EBM-2 culture medium (Lonza Group, Switzerland) at 25 °C on a DLS Malvern Zetasizer Nano ZS (Malvern, Spectris, London, UK). Fresh CuO-NPs and Ag-NPs stock solutions were always prepared in complete culture medium before toxicity tests; solutions were vortexed for approximately 1 min before being sprinkling onto cells to ensure homogeneity.

### Immortalized human cerebral microvascular endothelial cell (hCMEC/D3) culture

hCMEC/D3 it is a commercial cell line (Cedarlanelabs.com, CLU512, Ontario, Canada); cells are derived from human cerebral microvessel endothelial cells and are a stable, well-differentiated human BBB model (Weksler et al. 2005). Cells were cultured in EBM-2 medium (Lonza Group) supplemented with fetal bovine serum (5%), penicillin/streptomycin (0.5%), hydrocortisone (1.4 µM, Sigma-Aldrich) ascorbic acid (5 µg/mL, Sigma-Aldrich), chemically defined lipid concentrate (1/100, Life Technologies, Carlsbad, Ca, USA), 4-hydroxyethylpiperazineethanesulfonic acid (10 mM) (Life Technologies) and Human Basic Fibroblast Growth Factor (1 ng/mL) (Sigma-Aldrich). Cells were grown in 100 mm plates (Corning, NY, USA) pre-coated with rat type I collagen (R&D Systems, Minnesota, USA) and incubated at 37 °C in 5% CO_2_. The medium was changed every 2–3 days. For experimental procedures, cells were seeded in 96-well plates (Nunc, Denmark) at 5,000 cells/well, unless otherwise stated. Cell passages were performed using 0.25% trypsin (Gibco, Invitrogen, CA, USA) at 37 °C. For cell counting, cells were stained in 0.4% trypan blue and counted using a hemocytometer.

### Cytotoxicity assays

#### Tetrazole salt (EZ4U) reduction assays

EZ4U (Biomedica GmbH, Austria) reduction assays were used to evaluate the cytotoxic effects of CuO-NPs and Ag-NPs. For assays, 5,000 cells/well were seeded in 96-well plates (Nunc) and cultured for 24 h. Cells were exposed to CuO-NPs (5 – 400 µg/mL) or Ag-NPs (< 10 nm and < 40 nm) at 1–10 µg/mL. Control cells were grown in medium without NPs. Plates were incubated for 24 h or 72 h. Then, EZ4U assays were performed according to manufacturer's instructions. Briefly, the medium was removed and cells washed twice in fresh medium. Then, 200 µL medium and 20 µL substrate were added to wells and plates re-incubated for 3 h. After plates were shaken for approximately 5 min, the absorbance was read on a SYNERGY 2 microplate reader (BioTek Instruments, Inc.USA) at 450 nm/620 nm. Dye concentrations across samples were determined against samples without cells (blank). Cytotoxicity assays were performed in triplicate, IC_50_ values were determined.

#### Proliferation assays

For cell proliferation analysis, we used an IncuCyte S3 system (Live-Cell Analysis System, Sartorius, MI, USA). Cells were seeded into 96 well plates (Thermo Fisher) and grown for 24 h. Cells were then exposed to CuO-NPs (5 – 400 µg/mL) and Ag-NPs (< 10 nm and < 40 nm) at 1–10 µg/mL. After 24 h, NPs were removed by washing several times in fresh medium, plates placed in an incubator with an integrated IncuCyte fluorescence microscope, and allowed to warm to 37 °C for 30 min prior to scanning. Proliferation assessments (over 72 h) were conducted at least three times in independent experiments. Results were analyzed using Sartorius (MI, USA) software (v2018B).

#### Caspase 3/7 assays

Caspase 3/7 activity was evaluated using the aforementioned automated incubator IncuCyte S3 fluorescence microscope (Sartorius). Following manufacturer’s instructions, hCMEC/D3 cells were seeded in 96 well plates and grown for 24 h. Cells were then exposed to CuO-NPs (5 – 200 µg/mL) and Ag-NPs (< 10 nm and < 40 nm) at 1–10 µg/mL for 24 h or 48 h. As a positive control, cells were exposed to staurosporine (37 nM, Sigma-Aldrich). Then, medium was replaced with 100 µL fresh medium containing 1 × IncuCyte Caspase – 3/7 Apoptosis Assay Reagent (Sartorius) (final concentration = 5 μM). Plates were incubated in the Incucyte® Live-Cell Analysis System at 37 °C for 30 min prior to scanning. Fluorescence was measured using the fluorescein isothiocyanate (FITC) channel. At specific time points during automative microscopy, five images were taken/well in both brightfield and FITC channels. Green fluorescence (cleaved substrate) was measured at an excitation maximum wavelength = 500 nm and an emission maximum wavelength = 530 nm. Data were analyzed using Sartorius software (v2018B).

### Holotomographic microscopy

hCMEC/D3 cells were seeded into 35 mm culture dishes (IBIDI, Gräfelfing, Germany) at 20,000 cells/dish and incubated for 24 h. Then, cells were treated with 1 and 5 µg/mL Ag-NPs (< 10 nm and < 40 nm) or 5 and 25 µg/mL CuO-NPs for 24 h or 48 h. Fluorescent dyes were then added and samples incubated for 15 min at 37 °C to visualize cell structures. To visualize lysosomes, LysoView dye (1:1000, Biotium Inc. CA, USA) was used, while cell nuclei were stained with 4′,6-diamidino-2-phenylindole (1:1000, Sigma). ER staining was performed using 10 µM of 3,3′-dihexyloxacarbocyanine iodide (DiOC6 reagent (3), Biotium). Control (untreated cells) and exposed cells were incubated with dyes for 15 min at 37 °C and placed on the holotomographic microscope platform (3D Cell Explorer, Nanolive S.A. Switzerland) and three-dimensional tomographic images (z-stacks) generated. Background reduction and contrast enhancement post-processing steps were applied to images using STEVE software (Nanolive).

### Evaluating ER stress mediators and BBB dysfunction

#### RNA isolation and cDNA synthesis

Cells (3 × 10^5^ cells/mL/well) were incubated in 6-well culture plates (Techno Plastic Products, AG) for 24 h. Cells were then exposed to CuO-NPs (5, 25, 50 µg/mL) and Ag-NPs (< 10 nm and < 40 nm) at 1 and 5 µg/mL. After 24 h, the medium was removed, cells washed twice in PBS, and then suspended and lysed in RL buffer (EurX, Poland) plus 0.1% β-mercaptoethanol (Gibco, Invitrogen, CA, USA) for approximately 1 min. Cells were directly transferred to mini homogenization columns (EurX, Poland) in tubes and total RNA isolated using a GeneMATRIX Universal RNA Purification Kit according to manufacturer’s protocols (EurX, Poland), with optional on-column DNAse digestion. RNA samples in DNase/RNase free water were stored at –80 °C. RNA concentrations and quality were evaluated using a DeNovix DS-11 Spectrophotometer (DeNovix Inc. Wilmington, DE, USA). Next, 0.5 μg total RNA/reaction was reverse-transcribed to cDNA using an iScript Advanced cDNA Synthesis Kit for RT-qPCR (Bio-Rad Laboratories, Inc. CA, USA) using random hexamer primers according to manufacturer’s instructions. Experiments were performed on freshly isolated RNA from hCMEC/D3 cells from separate experiments, where each time a new total RNA pool was reverse transcribed and amplified.

#### Primer selection

The following databases were used to select primer sequences: Primer Blast and Gene Blast (National Center for Biotechnology Information), PrimerBank (public PCR primer resource) (Wang and Seed 2003; Spandidos et al. 2008, 2010). Several candidate primer pairs were selected for genes; amplicon lengths were determined and primer structural analyses performed using the OligoAnalyzer ™ Tool (Integrated DNA Technologies, IDT, IA, USA). Finally, primers were synthesized and purified by Genomed (Poland) on high performance liquid chromatography. Primer sequences are listed (Table 1).

**Table 1:** Study primers

Primer	Sequence 5′- > 3′:	Primer	Sequence 5′- > 3′:
GADPHf	GGAGCGAGATCCCTCCAAAAT	GRP78f	CATCACGCCGTCCTATGTCG
GADPHr	GGCTGTTGTCATACTTCTCATGG	GRP78r	CGTCAAAGACCGTGTTCTCG
IRE1af	AGAGAAGCAGCAGACTTTGTC	CHOPf	GAACGGCTCAAGCAGGAAATC
IRE1ar	GTTTTGGTGTCGTACATGGTGA	CHOPr	TTCACCATTCGGTCAATCAGAG
PERKf	GTCGCCAATGGGATAGTGACG	ERO1Bf	TTCTGGATGATTGCTTGTGTGAT
PERKr	GTCCGACAGCTCTAACAGTTTTT	ERO1Br	GGTCGCTTCAGATTAACCTTGT
ATF6f	AGCAGCACCCAAGACTCAAAC	DnaJB9f	TCTTAGGTGTGCCAAAATCGG
ATF6r	GCATAAGCGTTGGTACTGTCTGA	DnaJB9r	TGTCAGGGTGGTACTTCATGG
XBP1f	CCCTCCAGAACATCTCCCCAT	OCLNf	GACTTCAGGCAGCCTCGTTAC
XBP1r	ACATGACTGGGTCCAAGTTGT	OCLNr	GCCAGTTGTGTAGTCTGTCTCA
TJP2f	GGGAAGGTCGCTGCTATTGT		
TJP2r	CTCTCGCTGTAGCCACTCC		

#### qPCR

Assays were conducted using CFX Connect instrumentation (Bio-Rad) in a 96-well plate format, and transcripts quantitated using SsoAdvanced Universal SYBR Green Supermix (Bio-Rad). Reaction mixtures (final volume = 20 μL) included: 2 × SsoAdvanced Universal SYBR Green Supermix (Bio-Rad), reverse and forward primers (500 nM), cDNA (10 – 100 ng/reaction), and DNase/RNase-free water up to 20 µL. Amplification reactions included 40 steps of 95 °C for 10 s, then 60 °C for 20 s, with a final melt curve of 65 °C – 95 °C at 0.5 °C/5 s increments. Reactions were performed in triplicate. mRNA expression was normalized to endogenous control glyceraldehyde 3-phosphate dehydrogenase (GAPDH) mRNA levels and expressed as ratios between target and internal control gene mRNA levels using the comparative 2^−ΔΔCt^ method (Livak and Schmittgen 2001) .

#### Statistical analyses

Significant changes in mRNA expression levels, viability, caspase 3/7 activity were determined using Student's t-tests. *P* < 0.05 values were deemed statistically significant. Values were represented as the mean ± standard deviation (SD). Statistical analyses were carried out using Statistica, version 7.1.

## Results

### DLS measurements

**Fig. 1:**
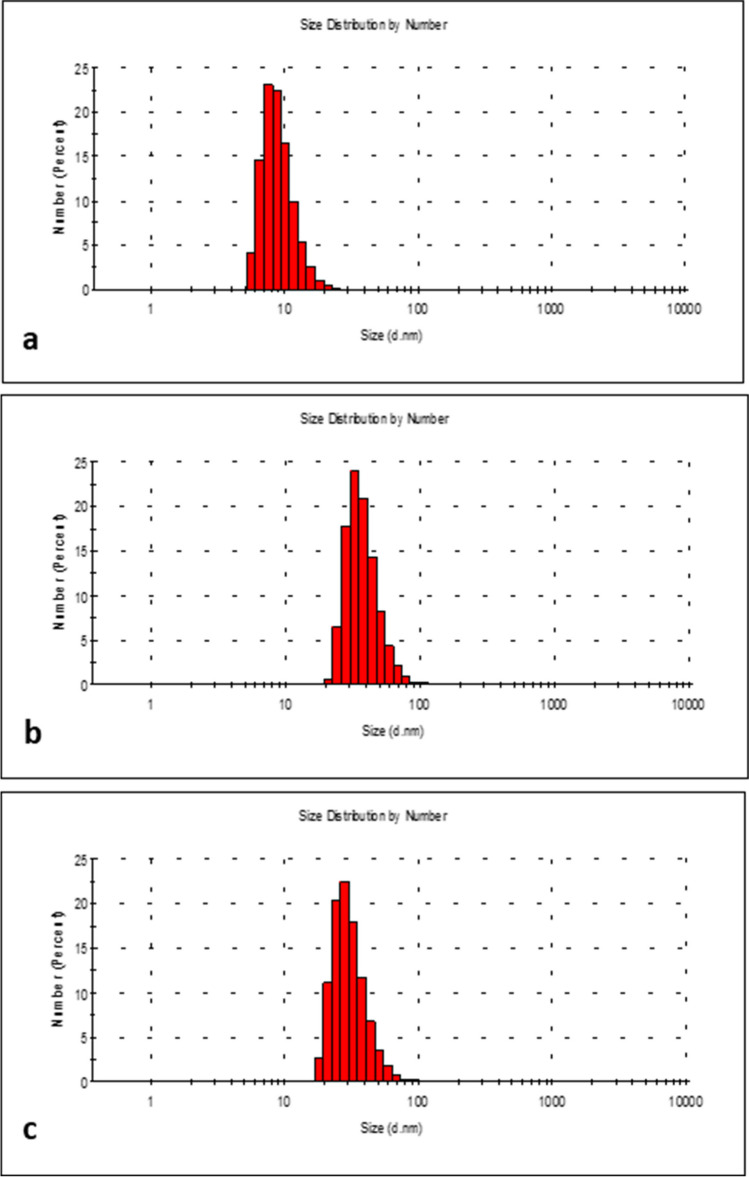
NP characterization. **a** Ag-NPs < 10 nm; **b** Ag-NPs < 40 nm; and (**c**) CuO-NPs < 50 nm

DLS measurements were made to assess NP sizes (Fig. 1.) and showed that 64% of all Ag-NPs (< 10 nm) were up to 10 nm (Fig. 1.a). For Ag-NPs (< 40 nm), 70% had diameters up to 40 nm (Fig. 1.b). Ag-NP (< 40 nm) suspensions were more homogeneous (polydispersity index (PDI) ≈ 0.178) when compared with Ag-NPs (< 10 nm) (PDI ≈ 0.483). DLS showed that 94% CuO-NPs (< 50 nm) (Fig. 1.c) had mean particle sizes of up to 50 nm, PDI ≈ 0.213.

### EZ4U cytotoxicity assays

#### Ag-NP EZ4U assays

**Fig. 2:**
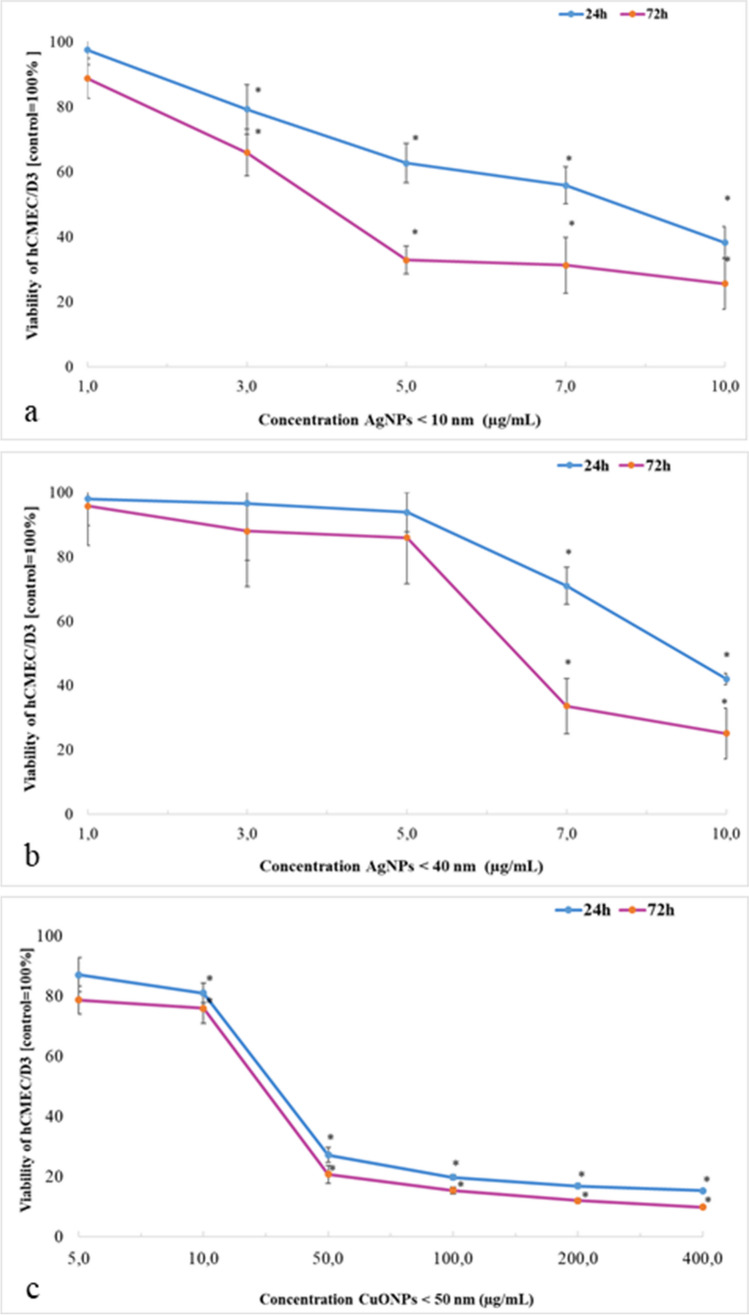
Cell survival assays. **a** Ag-NPs < 10 nm; **b** Ag-NPs < 40 nm; and (**c**) CuO-NPs < 50 nm. Results are represented by the mean ± SD. *: *P* < 0.05 (*n* = 3/group)

After 24 h and 72 h of hCMEC/D3 cell exposure to Ag-NPs (< 10 nm and < 40 nm), cell viability was tested using EZ4U assays (Fig. 2.). Cells showed greater sensitivity to Ag-NP (< 10 nm) toxic effects when compared with Ag-NPs (< 40 nm). A significant decrease in cell viability was observed at 3 µg/mL Ag-NPs (< 10 nm) at 24 h or 72 h exposure, which equated to 79.18% ± 7.65% and 65.89% ± 7.62%, respectively. The lowest viable cell percentage was observed after cell exposure to 10 µg/mL Ag-NPs (< 10 nm) for 24 h (38.22% ± 4.85%) and 72 h (25.59% ± 7.85%). For Ag-NPs (< 10 nm), the IC_50_ value after 24 h exposure was 8 µg/mL and after 72 h, 4 µg/mL (Fig. 2.a). For Ag-NPs (< 40 nm), cell survival decreased significantly after exposure to 7 µg/mL for 24 h or 72 h; 71% ± 5.7% and 33.58% ± 8.63%, respectively. Increasing Ag-NP (< 40 nm) concentrations to 10 µg/mL further decreased cell viability to 42% ± 1.85% and 25.06% ± 7.85% after 24 h and 72 h, respectively. For Ag-NPs (< 40 nm), IC_50_ values were 9.6 µg/mL after 24 h and 6.4 µg/mL after 72 h (Fig. 2.b).

#### CuO-NP EZ4U assays

A significant decrease in cell viability was observed at 10 µg/mL CuO-NPs after 24 h or 72 h exposure; 80.92% ± 3.30% and 75.95% ± 5.01%, respectively. The lowest viable cell percentage was observed after exposure to 400 µg/mL CuO-NPs (< 50) nm for 24 h (15.32% ± 0.68%) and 72 h (9.85% ± 0.33%). For CuO-NPs (< 50 nm), the IC_50_ value after 24 h exposure was 37.9 µg/mL and after 72 h it decreased to 31.12 µg/mL (Fig. 2.c).

#### Proliferation assays

**Fig. 3:**
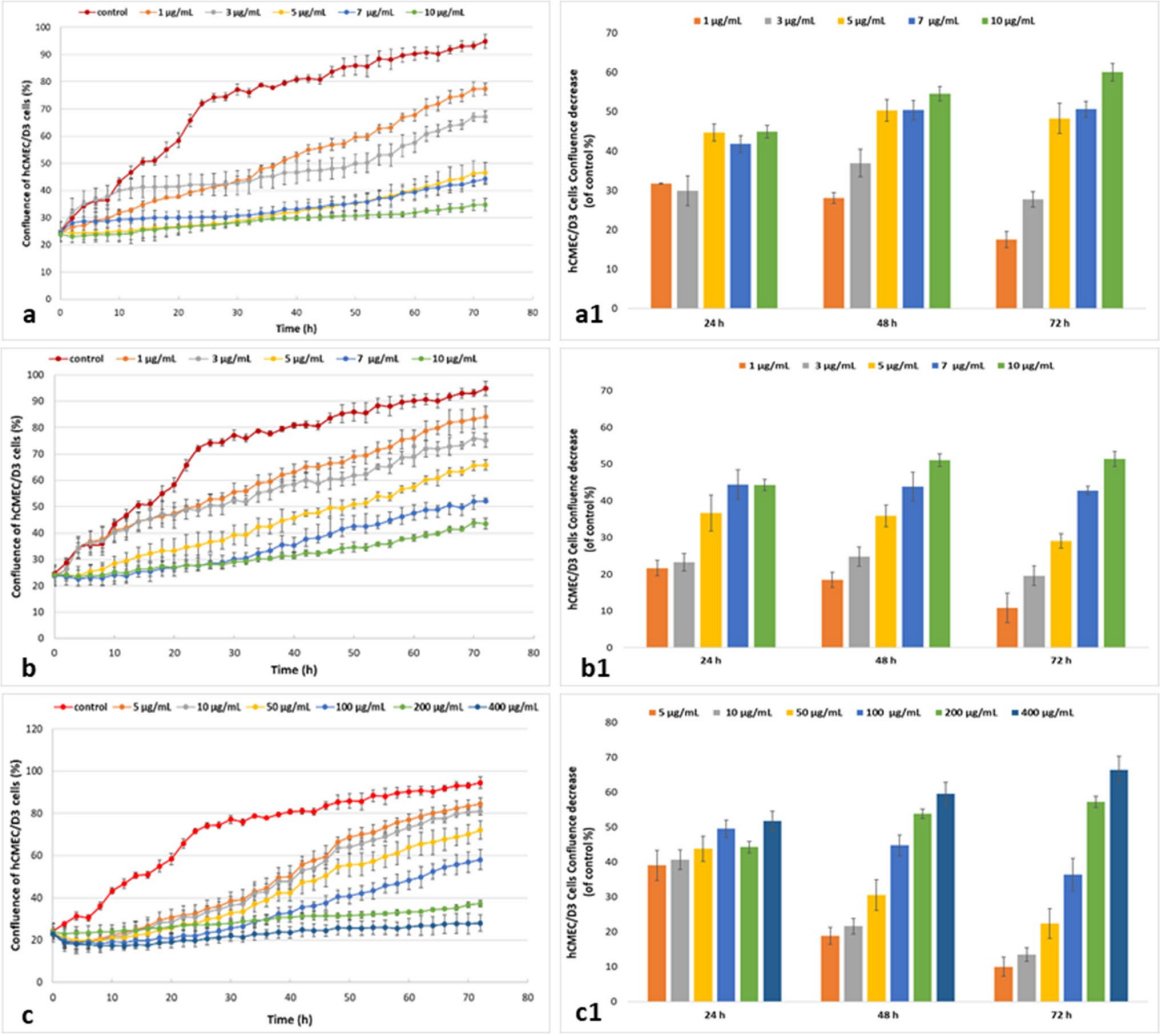
Cytotoxicity assay results; proliferation assays**. a –a1** Ag-NPs < 10 nm; **b-b1** Ag-NPs < 40 nm (**c-c1**) CuO-NPs < 50 nm

Percentage cell proliferation reductions, when compared with control hCMEC/D3 cells at all three time points, are shown (Fig. 3.). After 72 h exposure to Ag-NPs (< 10 nm) at 1, 3, 5, 7 and 10 µg/mL, decreased culture area coverage (percentage) relative to control cells was observed: 17.55% ± 2.1%, 27.75% ± 1.98%, 48.33% ± 3.81%, 50.67% ± 2.01%, and 60.07 ± 2.26%, respectively. Critically, only 1 µg/mL Ag-NPs (< 10 nm) at 24 h inhibited cell proliferation by approximately 31.75% ± 0.08% (Fig. 3. a-a1). Cell exposure to Ag-NPs (< 40 nm) at 1, 3, 5, 7 and 10 µg/mL decreased cell confluence by approximately 10.85% ± 4.04%, 19.64% ± 2.69%, 29.11% ± 1.95%, 42.80% ± 1.12%, and 51.38% ± 2.08%, respectively (Fig. 3. b-b1).

After 72 h, CuO-NPs (400 µg/mL) inhibited hCMEC/D3 proliferation relative to control cells by approximately 66.43% ± 3.84%, while CuO-NPs (200 µg/mL) over the same period inhibited cell proliferation by approximately 57.21% ± 1.55%. For 100 µg/mL CuO-NPs, inhibition was 36.4% ± 4.66%, at 50 µg/mL it was 22.4% ± 4.28%, and for 10 and 5 µg/mL CuO-NPs, proliferation was inhibited by 13.48% ± 1.89 and 10.00% ± 2.78%, respectively (Fig. 3. c-c1). Thus, hCMEC/D3 cells showed greater toxicity to Ag-NPs (< 10 nm) when compared with Ag-NPs (< 40 nm) and CuO-NPs (< 50 nm).

#### Caspase 3/7 assays

**Fig. 4:**
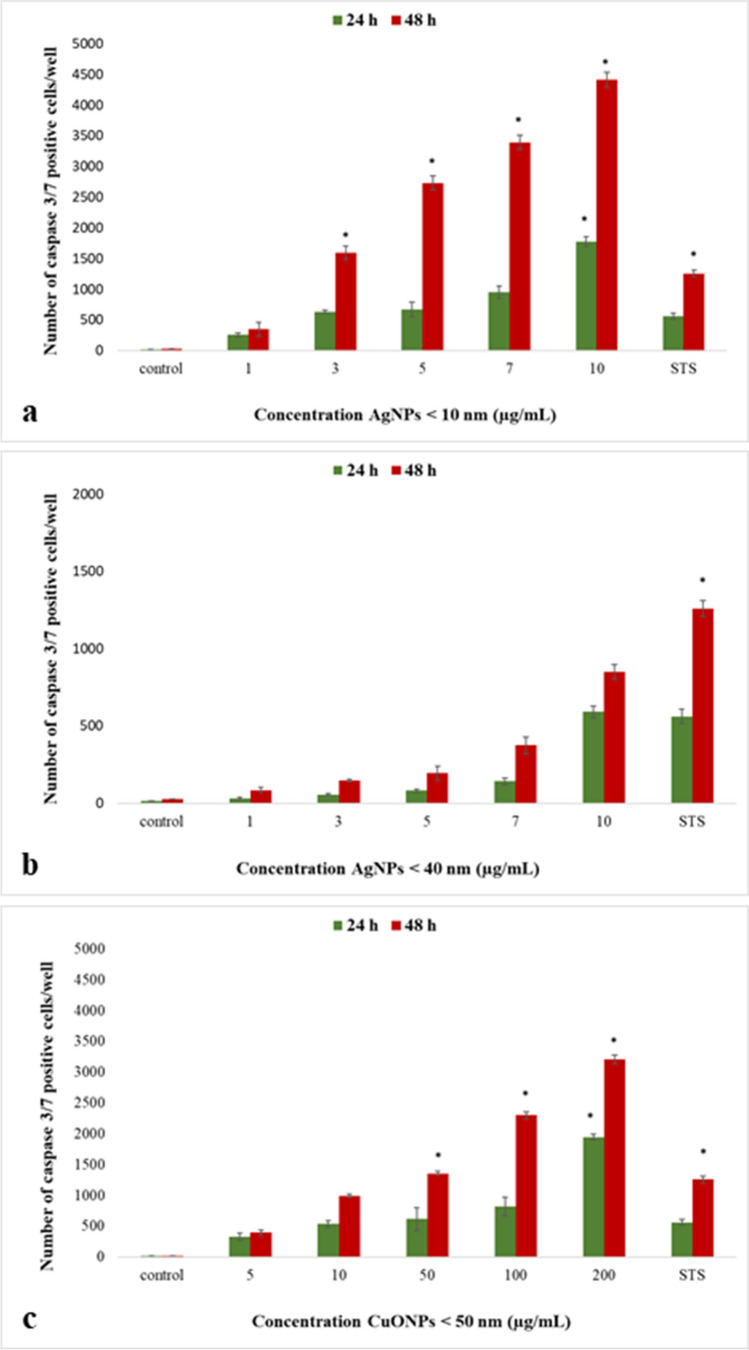
Cytotoxicity assays results; caspase 3/7 activity in hCMEC/D3 cells. **a** Ag-NPs < 10 nm; **b** Ag-NPs < 40 nm; and (**c**) CuO-NPs < 50 nm. Results are represented by the mean ± SD. *: *P* < 0.05 (*n* = 3/group)

We determined caspase 3/7 activity in hCMEC/D3 cells using the IncuCyte S3 system after 24 h or 48 h exposure to Ag-NPs (< 10 nm) and Ag-NPs (< 40 nm) at 1 – 10 µg/mL, CuO-NPs at 5 – 200 µg/mL, and 37 nM staurosporine (positive control). We observed dose- and time-dependent increases in apoptotic cell numbers (Fig. 4.). At 24 h in negative (untreated) and positive control cells, apoptotic hCMEC/D3 cells numbered 13.33 ± 0.20 and 560.93 ± 45.96, respectively, and after 48 h; 20.66 ± 0.41 and 1256.66 ± 52.07 cells were recorded, respectively. Ag-NPs (< 10 nm) significantly increased apoptotic cell numbers after 24 h at 10 µg/mL (1771.73 ± 78.57 caspase positive cells), and after 48 h exposure, 3 µg/mL generated 1593.13 ± 111.79 caspase positive cells (Fig. 4. a). When compared with untreated negative control cells, apoptotic hCMEC/D3 cell numbers were significantly increased after 24 h by CuO-NPs at 200 µg/mL; up to 1943.3 ± 43.4 cells (Fig. 4. c). Thus, caspase 3/7 activity in hCMEC/D3 cells was significantly dependent on exposure to 50 µg/mL CuO-NPs for 48 h (1355.4 ± 35.49 caspase positive cells).

### ER stress markers and BBB dysfunction

**Fig. 5:**
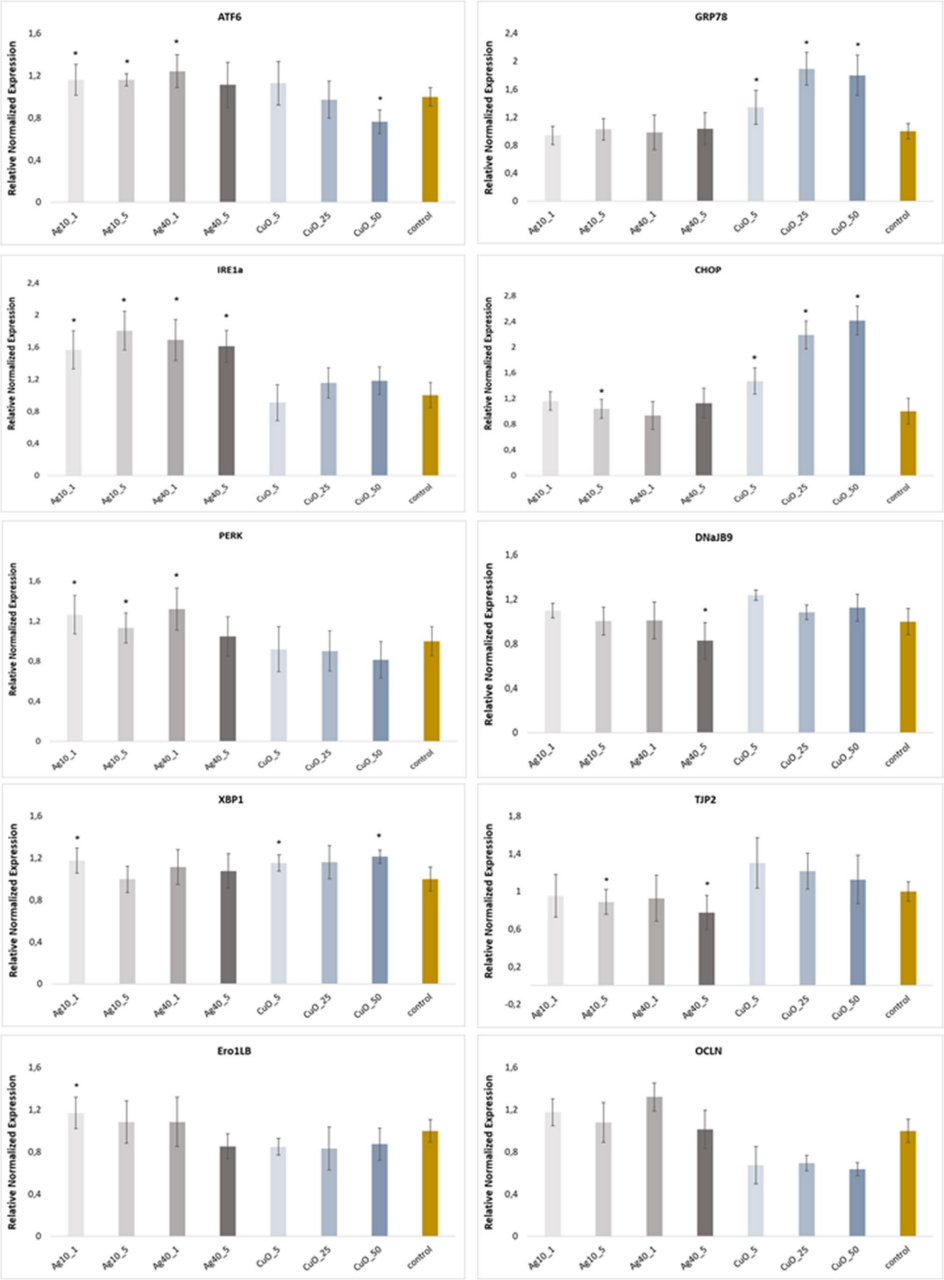
Relative normalized mRNA expression as a results of treating NPs. The mRNA levels for ATF6, IRE1a, PERK, XBP-1, Ero1LB, GRP78, CHOP, DnaJB9, TJP2 and OCLN are shown. Data were compared to values of GAPDH (reference gene) and then normalized with respect to the corresponding values at control and calculated using the 2^–∆∆Ct^ method. Results are represented by the mean ± SD. *: *P* < 0.05 (*n* = 3/group)

We examined the mRNA levels of several ER stress sensors (IRE1a, PERK, ATF6, XBP-1, GRP78, CHOP, Ero1LB, and DnaJB9) in hCMEC/D3 cells exposed for 24 h to NPs (Fig. 5.). IRE1a fold change levels increased after cell treatments with Ag-NP (< 10 nm and < 40 nm) at 1 i 5 µg/mL e.g. Ag-NPs < 10 nm at 5 µg/mL- 1.8-fold. Also, cells exposed to Ag-NPs (< 10 nm) at 1 µg/mL had significantly increased XBP-1 mRNA levels over 24 h. Additionally, XBP-1 mRNA levels were not significantly increased in cells treated with Ag-NPs < 10 nm at 5 µg/mL for 24 h. Thus, cells exposed for 24 h to Ag-NPs < 40 nm at 1 µg/mL and 5 µg/mL had increased XBP-1 mRNA levels. mRNA analyses in cells exposed to CuO-NPs at 25 µg/mL showed increased XBP-1 mRNA levels, while 5 µg/mL and 50 µg/mL concentrations significantly increased levels after 24 h when compared with untreated controls (Fig. 5.).

Therefore, we investigated other UPR expression patterns to examine if they were affected by different Ag-NPs and CuO-NPs related to hCMEC/D3 ER stress.

Increased ATF6 expression was observed in cells after exposure to 1 i 5 µg/mL Ag-NPs (< 40 nm and < 10 nm). We observed higher ATF6 expression after treatment with CuO-NPs at 5 and 25 µg/mL after 24 h compared with untreated controls. CHOP (CCAAT-enhancer-binding protein homologous protein) transcription factor mRNA levels were significantly increased by CuO-NPs treatment at 5, 25, and 50 µg/mL (1.47-, 2.18- and 2.41-fold, respectively). CHOP mRNA levels were significantly increased only for Ag-NPs < 10 nm at 5 µg/mL and were possibly induced by combined ATF6 and PERK pathways. In contrast, PERK, which is induced by misfolded protein-mediated ER stress, was increased significantly by Ag-NPs < 10 nm at 1 and 5 µg/mL and Ag-NPs < 40 nm at 1 µg/mL. In addition, increased GRP78 (BiP) levels were detected in cells after 24 h exposure to CuO-NPs at all concentrations (Fig. 5.).

UPR expression patterns involving Ero1LB and DnaJB9 factors were also analyzed. The DnaJ heat shock protein family (Hsp40) member B9 (DnaJB9) protein is located in the ER, induced by ER stress, and protects stressed cells from apoptosis (Lai et al. 2012), while Endoplasmic Reticulum Oxidoreductase 1 Beta (Ero1LB) is activated in ER membranes and facilitates ER protein folding (Ponsero et al. 2017). No significant changes were observed in DnaJB9 and Ero1LB expression levels, however, DnaJB9 was modestly upregulated at 24 h after CuO-NP treatment at 5, 25, 50 µg/mL and for Ag-NPs < 10 nm at 1 µg/mL. Similarly, Ero1LB was moderately upregulated after Ag-NP < 10 nm treatment.

Moreover, Ag-NPs (< 10 nm and < 40 nm) in cells treated with 5 µg/mL showed the expected significantly altered TJP2 expression (0.8- and 0.7-fold, respectively) (Fig. 5.)

### Morphological organelle evaluations

**Fig. 6:**
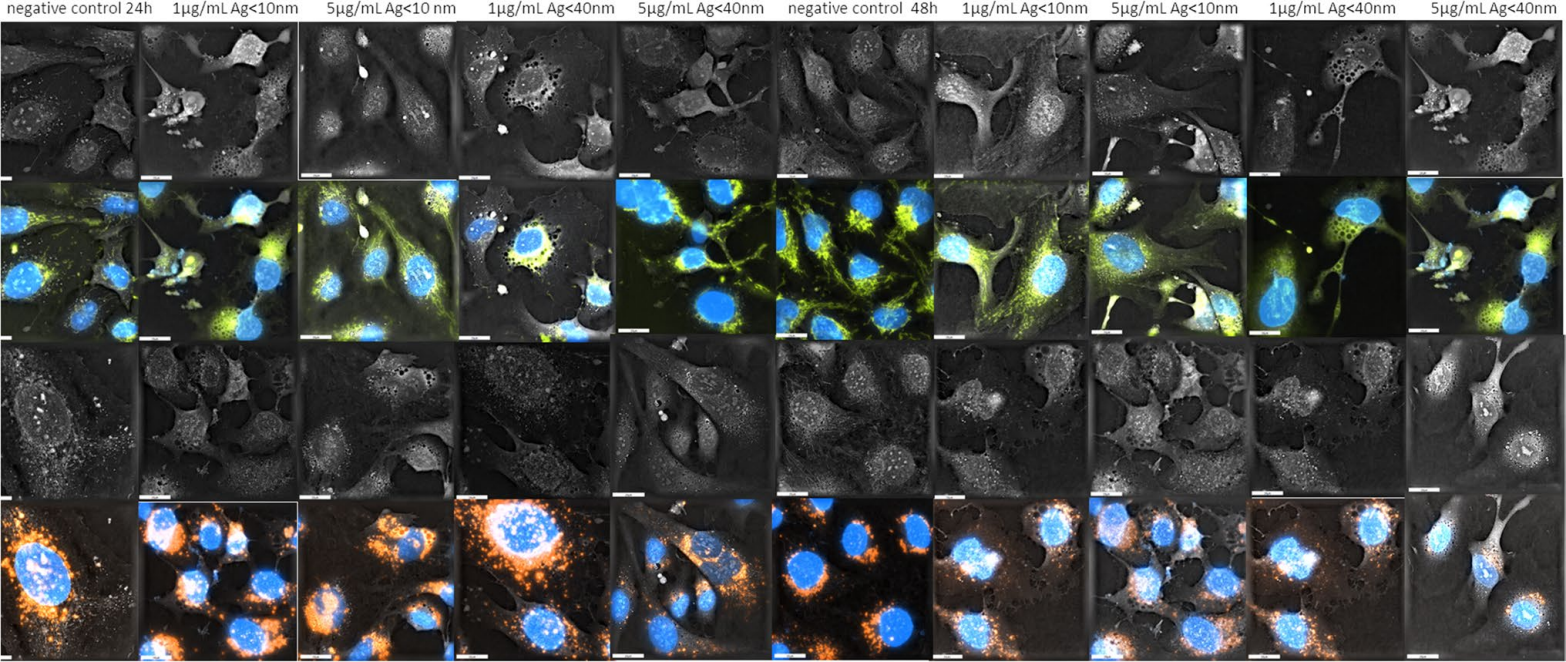
Holotomographic microscopy images showing morphological alterations in hCMEC/D3 cells exposed to Ag-NPs (< 10 nm and < 40 nm) (1 or 5 µg/mL) for 24 h and 48 h. Cells are shown in the bright field and after excitation with appropriate fluorochromes. HTM cell images show blue nuclei, orange lysosomes, and green ER structures. Scale bar = 20 µm

**Fig. 7:**
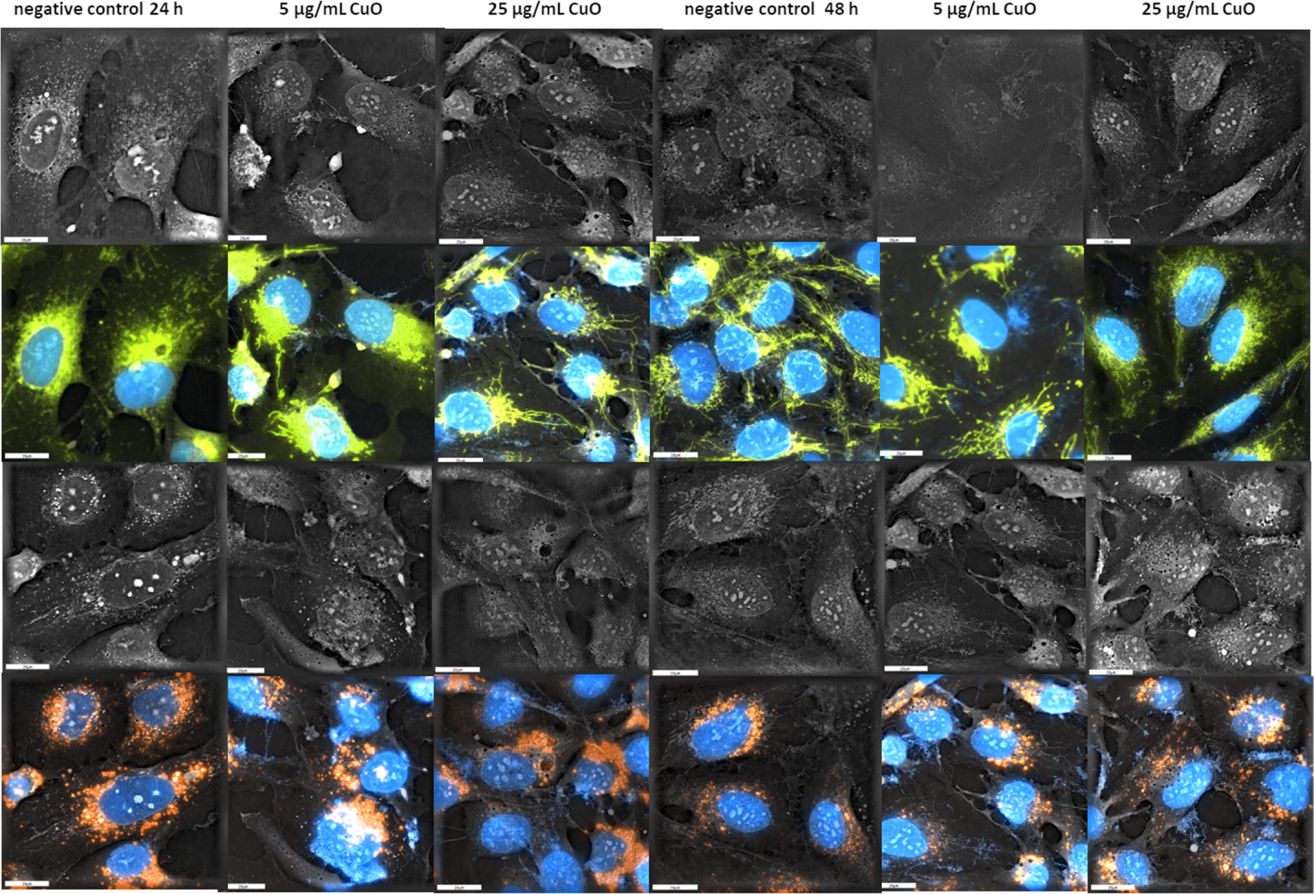
Holotomographic microscopy images showing morphological alterations in hCMEC/D3 cells exposed to CuO-NPs (5 or 25 µg/mL) for 24 h and 48 h. Cells are shown in the bright field and after excitation with appropriate fluorochromes. HTM cell images show blue nuclei, orange lysosomes, and green ER structures. Scale bar = 20 µm

Morphological changes were observed after hCMEC/D3 cell exposure to 1 and 5 µg/mL Ag-NPs (< 10 nm and < 40 nm) (Fig. 6.) and 5 and 25 µg/mL CuO-NPs (< 50 nm) (Fig. 7.) for 24 h and 48 h. Untreated cells (controls) showed no morphological or membrane damage; the ER was concentrated near the nucleus and homogenously distributed throughout the cytoplasm. Upon exposure to selected compounds, cell shape and size alterations were observed, including surface folding and changes in the vacuolar system. When compared with control cells, increased lysosome numbers and associated swelling were observed in the cytoplasm of exposed cells. At longer exposure times, cell areas became significantly decreased in CuO-NP and Ag-NP treatment groups. After staining, ER vacuolization and expansion were occasionally observed in cells, with ER structures enlarged and swollen (Figs. 6 and 7).

## Discussion

Evidence for DNA destruction and toxicity induction in BBB cells caused by metals, including CuO-NPs, was previously described by Sawicki et al. (2019), while Ag-NP toxic reactions and mechanisms toward rat brain endothelium structures were examined by Wu et al. (2015). Therefore, a dearth of studies exists in this subject area, especially in an era when so many Ag- and CuO-NP biomedical applications are prevalent (Plackal Adimuriyil George et al. 2018; Nicolae-Maranciuc et al. 2022; Fan et al. 2021).

EZ4U assays were used to assess cytotoxicity in cells exposed to different Ag-NP (1 – 10 μg/mL) and CuO-NP concentrations (5 – 400 μg/mL) for 24 h and 72 h, and identified several cytotoxicity profiles. Treating cells with Ag-NPs (< 10 nm) at 3 μg/mL significantly reduced cell viability independent of time of exposure (Fig. 2. a). In turn, Ag-NPs (< 40 nm) caused significant decreases in survival only after exposing cells to 7 μg/mL (Fig. 2. b). Contrary to our findings, Khan et al. (2019) measured cytotoxic effects in hCMEC/D3 cells using the 3-(4,5-dimethylthiazol-2-yl)-5-(3-carboxymethoxyphenyl)-2-(4-sulfophenyl)-2H-tetrazolium assay (MTS) at 24 h and 48 h after Ag-NP exposure. Considered relatively safe up to 10 μg/mL, Ag-NPs (50 nm) did not alter cell viability in 24 h when compared with controls, however, a significant 20% viability decline was observed in Ag-NP treatments for 48 h. Thus, particle aggregation at higher concentrations may have reduced cell viability. In our study, we determined IC_50_ values for Ag-NPs (< 10 nm) after 24 h exposure, and showed that the IC_50_ value was 8 µg/mL and after 72 h, only 4 µg/mL. For Ag-NP (< 40 nm) treatments, IC_50_ values were 9.6 µg/mL after 24 h and 6.4 µg/mL after 72 h. In contrast, Mishra et al. (2016) investigated human hepatoma HepG2 cells after 24 h exposure to 10 nm, 50 nm, and 100 nm Ag-NPs and showed that IC_50_ values were 5.1, 7.6, and 6.4 μg/mL, respectively. The authors observed no toxicity at lower Ag-NP concentrations (0.01 – 5 μg/mL) at any incubation time and no significant reductions in cell viability were observed in Ag-NP treatment groups (50 nm and 100 nm) at two time points after 6 h and 12 h exposure. Moreover, using cell viability data and IC_50_ values, doses ranging from 1 – 5 μg/mL and exposure times of up to 18 h were selected to assess molecular responses.

In our study, CuO-NP treatments (50 μg/mL) reduced cell viability to approximately 70%, after which no further decreases in cell viability were observed, despite exposure to higher concentrations. The IC_50_ value after 24 h exposure was 37.9 µg/mL, and after 72 h, it dropped to 31.12 µg/mL (Fig. 2. c). Similarly, Niska et al. (2015) assessed CuO-NP effects on immortalized mouse hippocampal cells (HT22 cells) using 3-(4,5-dimethylthiazol-2-yl)-2,5-diphenyltetrazolium bromide assays. Incubation with 1 – 80 µg/mL CuO-NPs for 6 h, 18 h, and 24 h generated a concentration- and time-dependent decrease in MTT-related cell viability. The IC_50_ after 24 h incubation was 25.9 µg/mL. Lian et al. (2017) used resazurin reduction assays to assess astrocyte and primary brain microvascular endothelial cell (BMEC) viability at 3 h, 12 h, 24 h, 36 h, and 48 h after CuO-NP exposure and showed that 1.5 mg/mL and 0.75 mg/mL CuO-NPs elicited the highest toxicity levels. Both BMECs and astrocytes treated with CuO-NPs for 24 – 48 h showed similar IC_50_ values, with a slight time-dependent toxicity increase. Hajizadeh et al. (2023) used different green biosynthesized CuO-NP concentrations (2.5 – 100 μg/mL) in human embryonic kidney cells to examine cellular effects for 24 h, 48 h, and 72 h. From MTT data, cell survival rates were over 50% at all-time points. The authors synthesized CuO-NPs using propolis extracts, which are non-toxic, environmentally friendly, and easy-to-use materials for CuO-NP synthesis. This innovative approach effectively reduced NP cytotoxicity and was relatively safe up to 100 μg/mL, thus exemplifying a 'safe by design' approach.

In several studies, Ag- and CuO-NPs were shown to influence proliferation in different cell types (Rodríguez-Razón et al. 2018; Shafagh et al. 2015). Smaller Ag-NPs were more toxic than larger NPs, in spite of the fact that smaller NPs tended to aggregate during cell uptake, which limited binding to cell membranes (Katarzynska-Banasik et al. 2021). In our study, at 24 h, 48 h, and 72 h, Ag-NPs, regardless of size, inhibited cell proliferation when compared with untreated cells (Fig. 3. a-a1, b-b1). AshaRani et al. (2009) verified that Ag-NPs and Ag^+^ ions released from NPs were involved in cell signaling cascades, and upon Ca^2+^ activation, catabolic enzymes were activated and damaged mitochondrial membranes. Moreover, they showed that signaling cascades have crucial roles in cytoskeleton deformation and inhibit cell proliferation. Our results suggested that in the first 24 h – 48 h, NPs at different concentrations elicited the greatest inhibitory effects on proliferation rates. Also, CuO-NPs induced anti-proliferation processes in cells (Fig. 3. c-c1) and induced higher proliferation levels when compared with untreated control cells after 24 h. Sun et al. (2012) reported that CuO-NPs caused not only oxidative stress, but also damaged mitochondria and demonstrated higher cytotoxic potential when compared with other metal oxide NPs.

Increased cytotoxicity which decreased cell proliferation was possibly caused by several different factors, ranging from NP content to size. A key cytotoxic NP effect is caspase activation and subsequent apoptosis effects (Parrish et al. 2013; García de la Cadena and Massieu 2016). Caspases are proteases which activate and implement apoptosis. In our study, significant caspase3/7 activation occurred at 10 µg/mL Ag-NPs (< 10 nm) at 24 h and 3 µg/mL Ag-NPs (< 10 nm) at 48 h. We also observed intensification of the apoptotic effect in hCMEC/D3 cell numbers after 48 h exposure to CuO-NPs doses from 50 µg/mL. Several studies have reported caspase activation during apoptosis induction; both caspase 3 and caspase pathways were activated in different mammalian cells, including BBB cells (Alarifi et al. 2013; Rakkar and Bayraktutan 2016; Yang et al. 2017; Glushakov et al. 2018; Lossi et al. 2018;). Our caspase 3/7 data showed that Ag- and CuO-NPs induced caspases in treated hCMEC/D3 cells, the activity of which increased in a concentration- and time-dependent manner (Fig. 4.).

BBB disturbances caused by outside interactions can affect the ability of endothelial cells to protect the brain (Pong et al. 2020; Wei et al. 2023). From a research perspective, any metal NP interactions with BMECs are critical considering that brain endothelial cells form complicated tight barriers between blood and brain tissue. In 2009, Choi and Zheng (2009) reported that Cu was transported to the brain as free Cu ions; the authors showed that high Cu accumulation occurred in brain barrier tissue and Cu transport to the brain occurred primarily via the BBB. Recent studies reported that toxic heavy metal NPs disrupted ER homeostasis and caused UPR induction by inducing ER stress (Cao et al. 2017; Gong et al. 2018; Liang et al. 2018; Khan et al. 2020). Therefore, we investigated if Ag-NPs and CuO-NPs induced ER stress in hCMEC/D3 cells by examining mRNA changes in multiple ER-stress related markers, and showed that IRE1a, PERK, ATF6, XBP-1, GRP78, CHOP, Ero1LB, and DnaJB9 mRNA levels increased significantly in hCMEC/D3 cells after exposure for 24 h. Sicari et al. (2020) assayed PERK activity using gene mRNA and protein levels, including ATF4 and CHOP. CHOP is an early mediator and promotes apoptosis, and upon PERK activation, induces NRF2 transcription factor phosphorylation. We showed that CuO-NPs increased CHOP mRNA levels, eliminated the anti-apoptotic effects of BCL2 (apoptotic cell death regulator), and blocked its expression (Szegezdi et al. 2006). Liu and Tang (2020) reported that Cu-NPs caused ER stress in rat hepatocyte BRL-3A cells via the apoptosis pathway and CHOP activation. Li et al. (2018) investigated the human neuroblastoma SH-SY5Y cell line and reported that ER stress occurred in cells treated with Ag-NPs, in particular, mitochondrial homeostasis was disrupted and the mitochondrial apoptosis pathway induced. IRE1a is an unfolded protein sensor in the ER and contributes to the Ag-NP-induced intracellular signaling UPR pathway. The protein specifically mediates XBP-1 splicing and activation. In our study, we identified mRNA changes in both IRE1a and XBP1 transcripts, consistent with ER stress-signaling pathway data for Ag-NP-induced apoptosis by Zhang et al. (2012). Quan et al. (2020) investigated Ag-NPs which induced mitochondrial apoptosis in the human retinal pigment epithelial cell line ARPE-19, and found that this process was directly connected with the IRE1a signaling pathway. Mitochondrial apoptosis occurred simultaneously with disruptions in cell cycle and autophagy processes. In extensive studies, the authors confirmed that during Ag-NP-induced ER-stress induction, increased fold changes in phosphorylated (p)-IRE1α and p-PERK expression was observed. Yang et al. (2017) showed that Cu-NPs not only increased XBP-1, ATF6, and CHOP levels, but that ER stress promoted apoptosis in kidney carcinoma—A498 and anaplastic large T cell lymphoma—SR786O cell lines. The authors identified increased calpain-1 levels, which is another ER stress-mediated apoptosis controller, after Cu-NP treatment. We identified increased mRNA levels in cells exposed to Ag-NPs (< 10 nm and < 40 nm) (Fig. 5.), and that three main ER stress sensors—IRE1a, PERK, and ATF6 (Almanza et al. 2019)—were activated. Thus Ag- and Cu-NP treatment, to a small extent, promoted ER stress by interfering with ER functions in hCMEC/D3 cells, and not only disturbed cell metabolism, but caused cell death.

NPs in this study, particularly Ag-NPs (< 10 nm and < 40 nm), significantly altered TJP2 expression at 5 µg/mL (Fig. 5.). As reported by Lee (2015), epithelial barrier permeability depends on TJ organization. However, we observed that Ag-NPs (< 10 nm) and CuO-NPs did not significantly reduce OCLN and TJP2 mRNA levels in hCMEC/D3 cells, consistent with Anspach et al. (2016). These authors studied modified gold NP (Au-NP) effects on TJs in hCMEC/D3 cells. In our study, TJP2 and OCLN expression in cells exposed to CuO-NPs dropped below control values after 48 h exposure (Supplementary Material), so decreased TJP2 and OCLN mRNA levels after NP treatment should indicate a permeable barrier. These proteins function as TJ barrier components in both epithelial and endothelial cells and are required for correct TJ function in barrier lineage cells (Bennett et al. 2019).

We previously reported that holotomographic microscopy could be used to observe morphological cell arrangements after low NP dose exposure exposure (Sawicka et al. 2021; Zapor et al. 2022). Herein, our results suggested that Ag-NP and CuO-NP doses below IC_50_ values induced morphological changes and adverse events in cells. Thus, at a fairly low exposure dose to 1 µg/mL Ag-NPs and 5 µg/mL CuO-NPs, ER and cytoplasm changes occurred in hCMEC/D3 cells which caused disruption. Huang et al. (2020), indicated that after entering cells, metal nanostructures remained in lysosomes and were redirected and accumulated in mitochondria, the ER, and nucleus, where they induced morphological changes. In ER morphology imaging in living cells, fluorescent dyes have been successfully used and include the long-chain carbocyanins DiIC16(3) and DiIC18(3) (Terasaki et al. 1986). In our study, significant morphological changes were manifested as cytoplasm vacuolization and ER expansion and swelling in treatment cell groups, thus providing important insights on NP effects. NP effects were characterized by the time-dependent loss of characteristic endothelial cell shapes and as a consequence, cell – cell contact loss, while in many cells, marked changes in nucleus shape were recorded, especially at 5 µg/mL Ag-NP (< 40 nm) (Fig. 6.) and 25 µg/mL CuO-NP doses after 48 h (Fig. 7.). Presented changes were intensified in time depended manner.

Stress-induced apoptosis by NPs at the BBB requires different and innovative strategies to identify metal NP influences. In our study, Ag-NPs and CuO-NPs induced hCMEC/D3 cytotoxicity and apoptosis in a dose-dependent manner. Both NP types induced ER-stress in hCMEC/D3 cells by increasing IRE1a, PERK, and ATF6 levels, and decreasing TJ barriers as manifested by reduced OCLN gene expression. At low NP concentrations, hCMEC/D3 cell organelle were altered by holotomographic imaging. Moreover, Ag-NPs and CuO-NPs induced three main ER pathways and stress-induced apoptosis in hCMEC/D3 cells.

Our in vitro evidence of Ag-NP and CuO-NP-mediated toxicity in human BBB cells suggests that NPs of this type should be included in occupational risk assessments for future biomedical applications, also ER stress should be considered as an early biomarker in toxicological evaluation. By examining NP-mediated ER stress in human BBB cells, we have provided important mechanistic insights on NP-induced toxicity. Our study also provides guidance for future research on using NPs to modulate ER stress in different anti-disease strategies.” Furthermore, future work should examine NP modifications and different metal NPs using Real-Time PCR to acquire more timepoint data with a view to actively capturing NP-induced BBB changes.

## Supplementary Information

Below is the link to the electronic supplementary material.

Supplementary file1 Figure 8. Relative normalized OCLN and TJP2 expression as a results of 48 h treating CuO-NPs. Data were compared to values of GAPDH (reference gene) and then normalized with respect to the corresponding values at control and calculated using the 2^–∆∆Ct^ method. Results are represented by the mean ± SD. *: *P* < 0.05 (*n* = 3/group) (PNG 15 kb)
