# Supplementary figures and images for: Assessing cytotoxicity and endoplasmic reticulum stress in human blood–brain barrier cells due to silver and copper oxide nanoparticles
Source: J Appl Genet. 2024 Feb 9;66(1):87–103. doi: 10.1007/s13353-024-00833-8 (PMC11761835; doi:10.1007/s13353-024-00833-8)

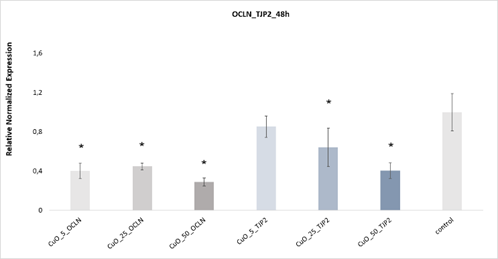

Supplement: Supplementary file 1 — Supplementary file1 Figure 8. Relative normalized OCLN and TJP2 expression as a results of 48 h treating CuO-NPs. Data were compared to values of GAPDH (reference gene) and then normalized with respect to the corresponding values at control and calculated using the 2–∆∆Ct method. Results are represented by the mean ± SD. *: P < 0.05 (n = 3/group) (PNG 15 kb) [file 13353_2024_833_MOESM1_ESM.png]
